# Supplementary material for: Palladium gates for reproducible quantum dots in silicon
Source: Sci Rep. 2018 Apr 9;8:5690. doi: 10.1038/s41598-018-24004-y (PMC5890286; doi:10.1038/s41598-018-24004-y)
Supplement: Supplementary file 1 — Supplementary Information [file 41598_2018_24004_MOESM1_ESM.pdf]

Palladium gates for reproducible quantum dots in silicon - Supplementary Information

Matthias Brauns[1,*], Sergey V. Amitonov[1], Paul-Christiaan Spruijtenburg[1], and Floris A. Zwanenburg[1]

[1]NanoElectronics Group, MESA+ Institute for Nanotechnology, University of Twente, P.O. Box 217, 7500 AE Enschede, The Netherlands
[*]matthias.brauns@ist.ac.at, present address: Institute of Science and Technology Austria, Am Campus 1, 3400 Klosterneuburg, Austria

ABSTRACT

Film morphology of Al and Pd films

For comparing the film morphology of deposited metal films, we prepared samples with PMMA on top with 200 nm wide lines patterned into the PMMA. We then evaporated Al and Pd under different pressure conditions and with different evaporation rates in the same electron-beam evaporator that is also used for the fabrication of the devices in the main text. This way, we simulate the conditions during device fabrication. In Supplementary Figure 1 we show SEM images of the as-deposited metal films. The Pd film in Figure 1d exhibits a small grain size of approximately 10 nm both on top of the PMMA as well as within the area of the patterned line. The Al films on the other hand have a much larger grain size on top of the PMMA leading to clogging of the patterned channel during evaporation. This is also the cause for the triangular cross section of the Al barrier gates in Figure 1 of the main text.

References

1. Mueller, F., Konstantaras, G., Spruijtenburg, P. C., van der Wiel, W. G. & Zwanenburg, F. A. Electron-Hole Confinement Symmetry in Silicon Quantum Dots. *Nano Lett.* **15**, 5336–5341 (2015). DOI 10.1021/acs.nanolett.5b01706.

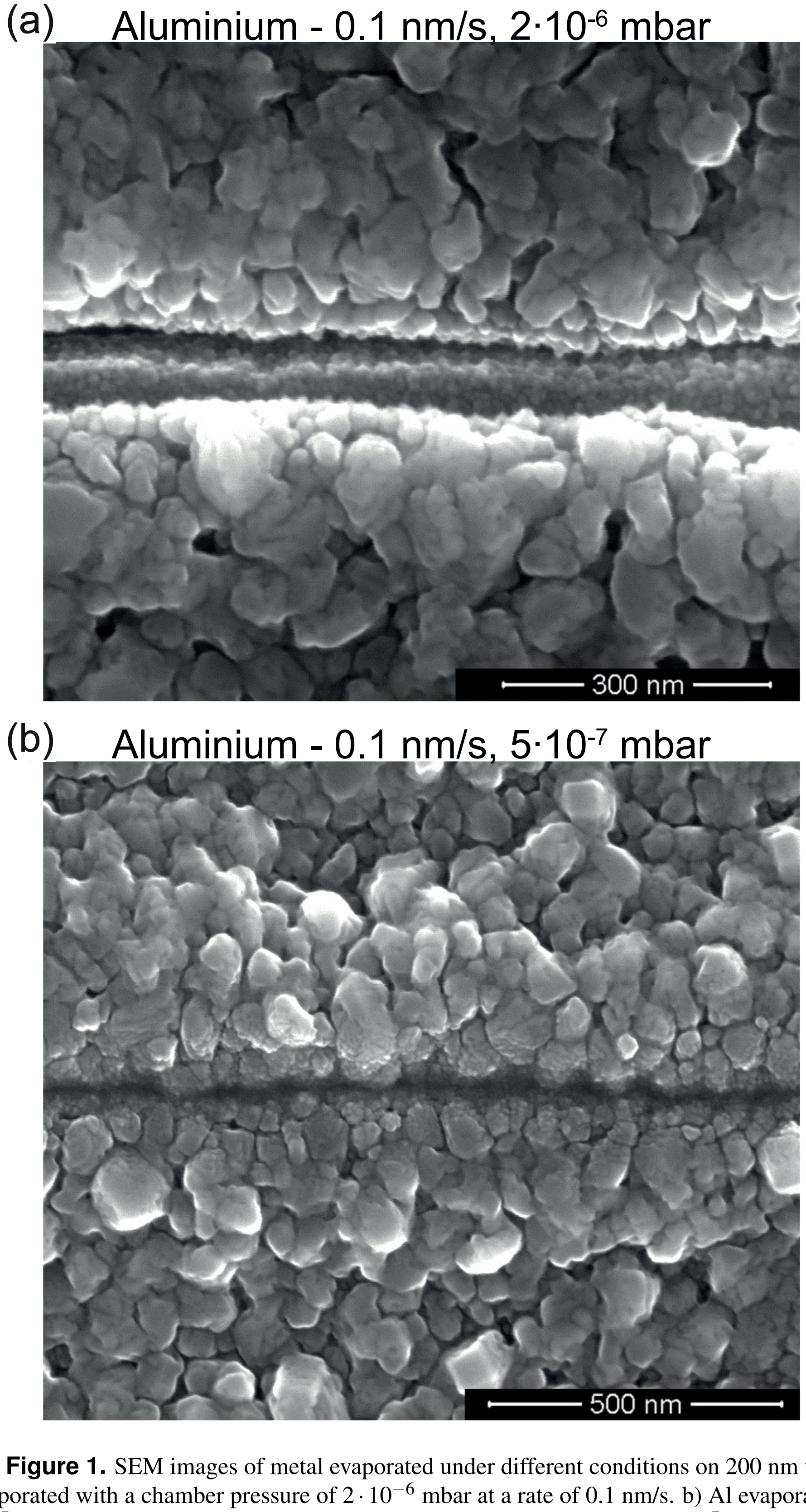

Supplementary Figure 1. SEM images of metal evaporated under different conditions on 200 nm wide lines patterned in PMMA. a) Al evaporated with a chamber pressure of $2 \cdot 10^{-6}$ mbar at a rate of 0.1 nm/s. b) Al evaporated with a chamber pressure of $5 \cdot 10^{-7}$ mbar, rate 0.1 nm/s. c) Al evaporated with a chamber pressure of $5 \cdot 10^{-7}$ mbar, rate 0.4 nm/s. d) Pd evaporated with a chamber pressure of $5 \cdot 10^{-7}$ mbar, rate 0.1 nm/s

(c) Aluminium - 0.4 nm/s, 5·10^{-7} mbar

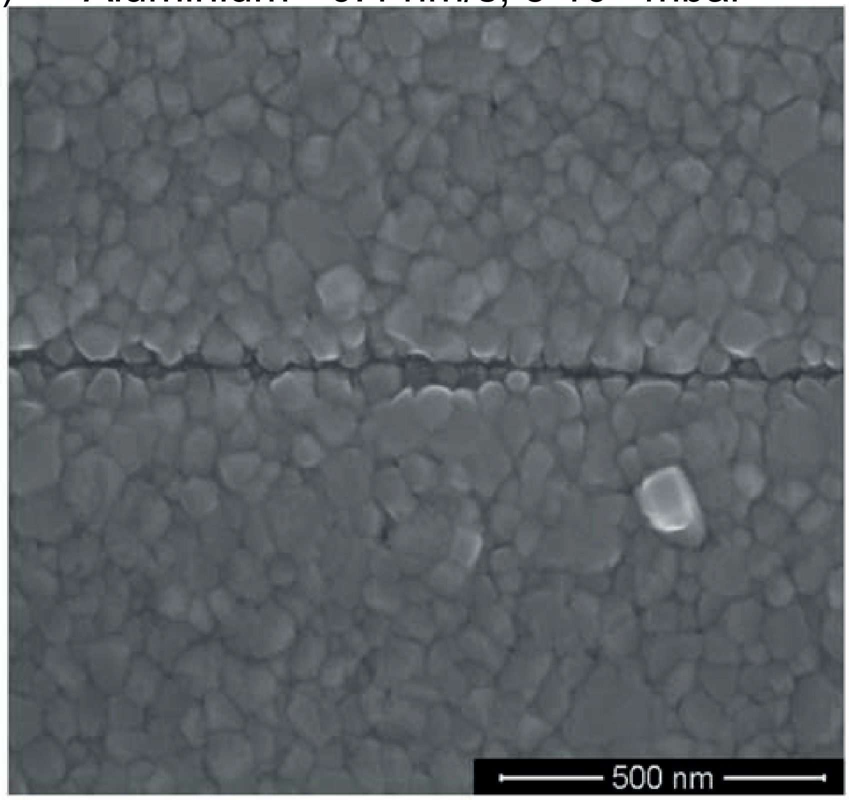

(d) Palladium - 0.1 nm/s, 5·10^{-7} mbar

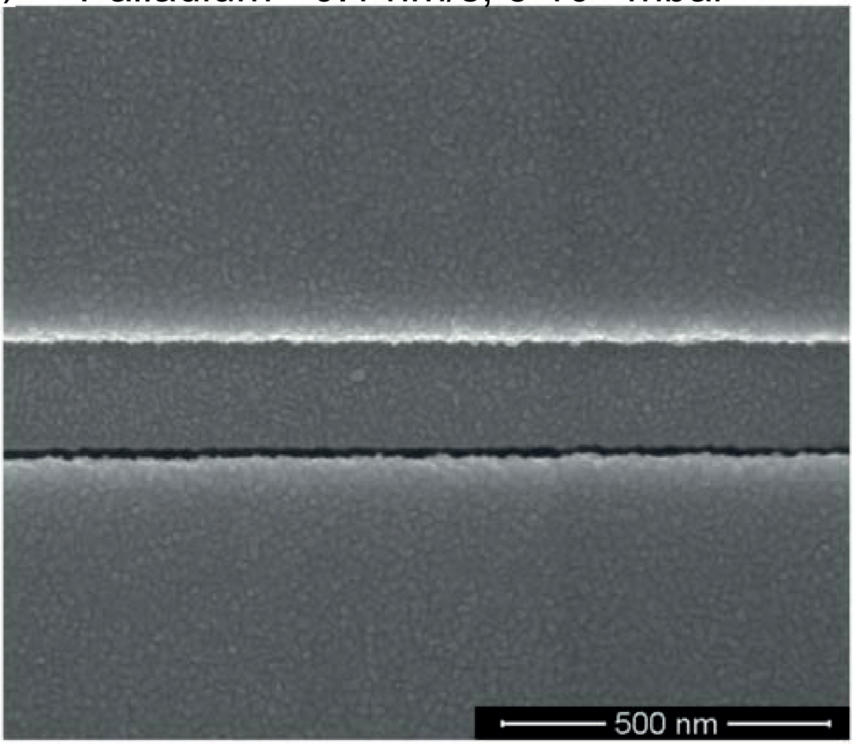

Supplementary Figure 1. SEM images of metal evaporated under different conditions on 200 nm wide lines patterned in PMMA. a) Al evaporated with a chamber pressure of $2 \cdot 10^{-6}$ mbar at a rate of 0.1 nm/s. b) Al evaporated with a chamber pressure of $5 \cdot 10^{-7}$ mbar, rate 0.1 nm/s. c) Al evaporated with a chamber pressure of $5 \cdot 10^{-7}$ mbar, rate 0.4 nm/s. d) Pd evaporated with a chamber pressure of $5 \cdot 10^{-7}$ mbar, rate 0.1 nm/s.
